# Involving research participants in a pan-European research initiative: the EPAD participant panel experience

**DOI:** 10.1186/s40900-020-00236-z

**Published:** 2020-10-15

**Authors:** S. Gregory, E. M. Bunnik, A. B. Callado, I. Carrie, C. De Boer, J. Duffus, K. Fauria, S. Forster, D. Gove, I. Knezevic, A. Laquidain, D. Pennetier, S. Saunders, S. Sparks, J. Rice, C. W. Ritchie, R. Milne

**Affiliations:** 1 Edinburgh Dementia Prevention, Centre for Clinical Brain Sciences, University of Edinburgh, Edinburgh, UK; 2 Department of Medical Ethics and Philosophy of Medicine, Erasmus MC, University Medical Centre Rotterdam, Rotterdam, The Netherlands; 3 BarcelonaBeta Brain Research Center, Pasqual Maragall Fundation, Barcelona, Spain; 4 Centre de Recherche Clinique du Gérontopôle, Toulouse University Hospital, Toulouse, France; 5 VUmc Alzheimercentrum, Amsterdam, The Netherlands; 6 Participant panel member, Barcelona, Spain; 7 Department of Psychiatry, University of Oxford, Oxford, UK; 8 Alzheimer Europe, Luxembourg, Luxembourg; 9 Institute of Public Health, University of Cambridge, Cambridge, UK; 10 Society and Ethics Research, Wellcome Genome Campus, Hinxton, UK

**Keywords:** Involvement, Cohort, Participant panel, At-risk, Readiness, European

## Abstract

**Background:**

**Including participants in** patient and public involvement activities is increasingly acknowledged as a key pillar of successful research activity. Such activities can influence recruitment and retention, as well as researcher experience and contribute to decision making in research studies. However, there are few established methodologies of how to set up and manage participant involvement activities. Further, there is little discussion of how to do so when dealing with collaborative projects that run across countries and operate in multiple linguistic and regulatory contexts.

**Methods:**

In this paper we describe the set-up, running and experiences of the EPAD participant panel. The EPAD study was a pan-European cohort study with the aim to understand risks for developing Alzheimer’s disease and build a readiness cohort for Phase 2 clinical trials. Due to the longitudinal nature of this study, combined with the enrolment of healthy volunteers and those with mild cognitive impairments, the EPAD team highlighted participant involvement as crucial to the success of this project. The EPAD project employed a nested model, with local panels meeting in England, France, Scotland, Spain and The Netherlands, and feeding into a central study panel. The local panels were governed by terms of reference which were adaptable to local needs.

**Results:**

The impact of the panels has been widespread, and varies from feedback on documentation, to supporting with design of media materials and representation of the project at national and international meetings.

**Conclusions:**

The EPAD panels have contributed to the success of the project and the model established is easily transferable to other disease areas investigating healthy or at-risk populations.

## Plain English summary

This paper reports on the set up, running and experiences of patient and public involvement in a European dementia prevention study. The European Prevention of Alzheimer’s Dementia (EPAD) project was set up to understand more about risks for developing dementia in people with no or mild symptoms, and to create a group of people who would be willing to join drug studies looking to prevent the development of dementia. Patient and public involvement was identified as an important part of EPAD from the outset as it asked for long term commitment from a group of people who were ‘at risk’ for a disease rather than having the disease of interest. To achieve this EPAD set up a series of ‘participant panels’ in England, France, Scotland, Spain and The Netherlands. These panels were formed from groups of participants in the study who met at least twice a year with researchers to provide feedback on their study experiences, study paperwork and contribute to study planning. The panels fed into a central study panel who met once a year for overall input into the study. The paper describes how participants were invited to join the panels as well as how the meetings were organised. Panels successfully contributed to study documents, study videos and presenting at national and international meetings. The panels were an important part of the success of the EPAD project and we suggest the set up would be easy to replicate in other studies.

## Introduction

The importance of patient and public involvement in health care research has been increasingly acknowledged over the last 25 years [1, 2]. Compared to participation or engagement, involvement means patients and the public taking an active role in the shaping of research in partnership with researchers. More recently, these ‘PPI’ activities have been complemented by new roles for participants themselves in shaping and guiding the development and conduct of studies.

Opportunities for public and patient involvement range from involvement in the commission and design of research ideas through to involvement in the active process of managing the research study as well as dissemination of findings. Review bodies such as the UK based Health Research Authority (HRA) and many funding bodies strongly encourage such PPI activities as part of a applications, and the INVOLVE network has been established in the UK to support integration of PPI into health and social research [3, 4]. However, while PPI initiatives are well established in the UK, there is less experience in many other European countries, although patient-run charities, for example, have been influential partners in setting research agenda, and recent initiatives such as EUPATI aim to develop opportunities for patient involvement in clinical trials and health technology assessment [5–8]. In the case in dementia research, a scoping review of European PPI activities in dementia research, found that they were concentrated in the UK, with 19 projects identified, compared with 1 from the Netherlands and none elsewhere [4]. Since then, a framework has been published by the MOPEAD consortium, based in Spain, focussed primarily on the recruitment and retention of participants [9].

The literature on PPI identifies four areas of impact; on the individual, the research, the researchers and societal benefit [10].PPI involvement has been linked to benefits in securing funding [11], modest effects on recruitment [12, 13] and an increased retention [14], as well as benefiting researchers [15] and funders [16]. The INVOLVE network have identified examples of real world benefit to studies incorporating PPI, with quality the overarching benefit influencing areas ranging from the relevance of the research topic to the implementation and impact of research [17]. Interestingly, a study in the primary care setting found researchers perceived the most positive impact of PPI when the approach included more indicators of good practice such as offering training to contributors [13]. However, despite the number of frameworks described, researchers often feel unequipped in knowing how best to involve patients and members of the public and what benefits they might reasonably expect [18].

While PPI in general is well established, and may involve long term partnerships between researchers and lay members, there is growing interest in including the perspective of those currently taking part in biomedical research on a systematic and ongoing rather than an ad hoc basis [19, 20]. Dillon et al. for example, in mapping the impact of patient engagement in research, describe the importance of anticipating participant issues, with the benefits described above, but do not consider potential active roles for participants themselves [21]. It has been suggested that the experience and expertise of participants directly affected by research activities as well as the wider public and patient community, can “contribute knowledge and ethical perspectives highly relevant to research decisions” [22] that can improve the success of the research project. Participant perspectives are thus being incorporated throughout the research process, ranging from decisions about data access [23] to the establishment of ‘participant panels’ in a range of longitudinal or large-scale studies, including the ALSPAC study [24], the UK’s 100,000 Genomes project [25] and the US *AllofUs* precision medicine initiative [26]. However, there has been little discussion of how such participant, patient and public involvement, or PPPI (3PI) can work, or what difference it can make.

This paper presents the experience of the European Prevention of Alzheimer’s Dementia (EPAD) programme with establishing and running panels of research participants across multiple European countries. EPAD was a pan-European initiative aiming to better understand the early stages and risks of developing Alzheimer’s disease, at the same time developing a platform to run innovative interventional trials to test new compounds [27, 28]. The project recruited over 2000 participants in 11 countries throughout Europe, with annual visits to the research centres to complete a range of physical and psychological assessments (with a 6 month visit in year 1 to evaluate cognitive function). Participants were seen in the longitudinal cohort study (LCS) for up to 4 years of clinic visits. Participants eligible to enrol in EPAD LCS were cognitively healthy or have mild cognitive impairment (MCI), were generally fit and well, had someone to attend as a study partner and were willing in principle to consider enrolment into a clinical trial of an investigational medicinal product.

One of the founding goals of the EPAD programme was to involve participants as research partners. As with much PPI activity, this was a result of multiple and overlapping motives [2](ref). it reflected a desire to learn from the experience of participants to improve the study, thereby increasing successful retention, but also a normative drive to reciprocate participants’ fundamental contribution to the project, and to ensure that those most directly affected by changes to the project were represented in discussion and decision-making.

Responsibility for planning participant involvement activities was identified at the outset of the project as a key task for the ethics workgroup in EPAD, and resources were allocated to the development of these activities at the outset of the project. This planning work built on learning from patient and public involvement work during the project setup with members of the public and patient groups [29], completed in collaboration with Alzheimer Europe and the European Working Group of People with Dementia, and building on the success of previous participant involvement activities [30, 31]. The scope of for potential participant input identified in this work included but was not limited to study design, understanding the research experience, input on communications and future planning.

In this paper we describe the approach to setting up both the country level and project wide participant panels, the impact participant involvement has had on the project and how the model developed in EPAD could be used by other research fields. The paper draws on the reflections on researchers and research participants on the experience of being involved in designing, setting up and participating in the panels. It draws on reports from study staff at each centre with a currently operating panel, study documents related to the panel developed by the EPAD ethics workgroup, and feedback from panellists in each local participant panel. This paper was written in line with the GRIPP2 guidelines, with additional information available in the GRIPP2 short form checklist [see additional file 1].

## Methods

We asked two sets of questions (one to researchers and one to participant panel members) to gather data on the set up, running and perceived impact of the panels. Researchers were asked to reflect on their experience of recruitment and set up, running of the panel (primarily focusing on logistical and operational issues), the content of the panel and the impact of panel discussions on the study so far. Panel members were asked a series of questions about their experiences and to reflect on the following areas: their expectations before joining the panel, in their words how does the panel they sit on operate, what do they think the panel has achieved and any other comments they wished to add. At least one researcher from each panel contributed to answering the questions about the panel and are included as co-authors on this paper. At least one panel member from each panel contributed answers. The Scottish and Spanish panels were able to circulate the questions to the entire panel and the panel member co-authors led on collecting and synthesising this data.

## Results

### The panel structure and the central panel

**Fig. 1:**
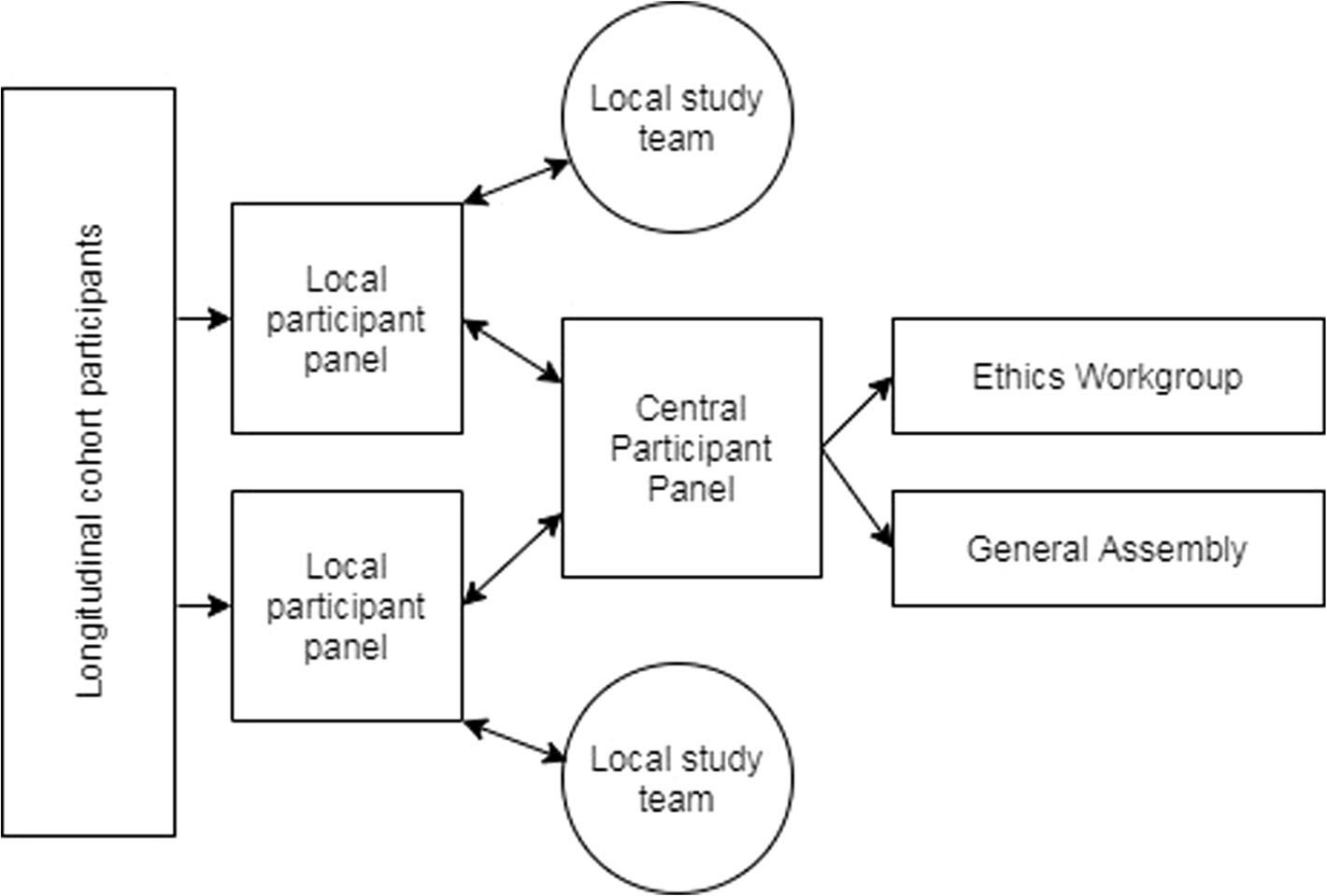
Overview of local and central participant panel set-up within the EPAD LCS study structure. Local panels were recruited from longitudinal cohort participants. They worked with local study teams, and were represented on the central participant panel. This central panel fed directly into the work of the EPAD ethics workgroup and the General Assembly of the project

The EPAD panel set-up drew upon the experience of the PREVENT Dementia Study [30]. Key features of the EPAD participants’ panels included a nested panel structure, in which multiple local panels function independently (Fig. 1). Nominated members from these local panels then formed a single study wide panel, termed the central panel. Nominations for membership to the central panel were by peers within the panel, with no staff involvement. This central panel met in 2018 and 2019, at the General Assembly of the project.. The central panel meeting was chaired by the EPAD Ethics group and was closed to other members of the consortium unless specifically invited by the panel members. The structure of each panel is described below, with an overview of the structure provided in Table 1 for comparison.

**Table 1:** Overview of local and central panel set-up arrangements in the EPAD LCS study

	Scotland	England	France	The Netherlands	Spain	Central panel
*Initial introduction and invite to panel*	Invite letter/email to all participants	Invite letter/email to all participants	Invite letter/email to all participants	Introduced during annual meeting	Selected participants contacted by telephone	Discussed by staff during local panel meetings
*Panel enrolment criteria*	Allocated on first come first serve basis	Allocated on first come first serve basis	Participants prioritised based on time in the study	Allocated on first come first serve basis	Allocated on first come first serve basis	Nominated by members of local panels
*Waiting list for new members in operation?*	Yes	Yes	Yes	No	Yes	No
*Chair*	Participant	Participant	Participant	Participant	Participant	Staff
*Vice-chair*	Role not established	Role not established	Participant	Role not established	Participant	Role not established
*Frequency of meetings*	Every 6 months	Every 6 months	Every 6 months	Every 6 months	Every 3 months	Once a year at General Assembly meeting
*Number of participants*	12	8	9	7	8	6–10
*Communication methods*	Face to face, post, email and telephone	Face to face, post, email and telephone	Face to face, email and telephone	Face to face and email	Face to face, post, email and telephone and WhatsApp	Face to face

#### Central panel

The central panel meeting had two main goals – to co-ordinate activities across the local panels, and to provide for direct participant input into the development of the study. Participation in the General Assembly provided the opportunity for participants to learn about the progress of the study, to input into the primary decision-making body of the consortium, and to provide feedback through both plenary and closed meetings. The latter were formally chaired by the EPAD ethics workgroup, although informal breakout meetings were also convened by panellists themselves to enable learning across different countries’ panel experiences.

Meetings of the central panel took place in 2018 and 2019, with six and ten members respectively attending both the panel meeting and the General Assembly. In addition, one participant representative attended the 2017 project General Assembly, at which point only the Scottish panel had been established. Consequently, no central panel was held during this meeting. During the central panel members could discuss items that had arisen during their local panels, raise topics for discuss and discuss agenda items introduced by the EPAD staff. Panel members then fed back on their experiences from the central panel and General Assembly attendance at local panel meetings.

In addition to meetings of the central panel, participant panel members have also contributed to the planning of the future direction of the project as it approaches the end of its initial funding. Participant panel members from two EPAD countries worked with the research team in planning for the sustainability of the project and the long-term use of EPAD samples and data.

#### Local panels

Each country was given a mandate to establish participant involvement on either a research centre or country level in the form of a panel. Whilst the common language spoken at the central panel was English, requiring a certain level of ability to speak English, the local panels facilitated multi-lingual involvement with participants. A terms of reference document was created as a guide for research teams (Appendix One), however these terms could be adapted as appropriate to meet local requirements and based on discussions with local panel members. This discussion was a key stage in establishing and managing the expectations of participants and researchers about the participant panel role. We describe below the set up and running of the panels with commonalities first described, followed by any unique adaptations made:

##### Scotland

In Scotland there was an established centralised country wide panel, with membership from four recruiting centres (NHS Lothian, Grampian, Greater Glasgow & Clyde and Tayside). The choice to form one country wide panel was advocated for by the participant members and worked well in a geographically small country. Towards the end of the project a participant led panel was also established in NHS Grampian. Panel members from Grampian advocated for this due to interest from their fellow participants in the area to contribute their feedback to the panel without joining the Scottish wide group.

##### England: Oxford, West London and Bristol

England similarly established a panel to represent participants from multiple centres. The panel ran from Oxford, England and involved participants from three centres (Oxford, West London and Bristol).

##### The Netherlands: Amsterdam

The panel in the Netherlands was housed at the VuMC (Vrije Univercentreit Medical Centre). As there was only one centre in the Netherlands this panel operated both as the country and centre wide panel.

##### France: Toulouse

France had one panel in operation, based and run from the Toulouse centre. As one of the largest centres in the EPAD study Toulouse was able to harness the participant voice onto this panel.

##### Spain: Barcelona

Spain’s panel was in Barcelona, the first EPAD centre to open in Spain.

### Establishing the panels

The panels were in operation for a range of time, with the Scottish and Barcelona based panels established in early 2017, and the newest panels, England and Toulouse, established in 2019. All panels have met at least twice at the time of this paper.

Panels employed a variety of recruitment methods during the initial set-up period, all of which were successful. Three panels (Scotland, England and Toulouse) contacted all local participants via letter or email to explain that a participant panel was being established and asking for interested participants to contact the coordinating centres to receive more information. In Amsterdam the panel was first introduced during an annual meeting for participants, to which all EPAD participants were invited, and the panel opportunity was followed up during the dissemination of minutes from this meeting. In order to maximise the engagement of the participants and the output of the panel, the team in Barcelona established a list of criteria for the selection of the potential panel members such: proximity to the centre, sex, age, English language level, motivation. These were participants who had previously expressed interest in being more involved in the study and each participant was contacted by phone to assess interest in joining the panel. Most panels enrolled people on a first come first served basis, with the exception of Toulouse which enrolled based on longevity in the EPAD study. At the time of the cohort study closing a waiting list was in operation at the Scottish, English, Toulouse and Barcelona panels due to levels of demand. New recruits were informed about the participant panel using flyers in Scotland and via email in Barcelona, whilst Amsterdam elected to maintain a static panel as the participants involved have the most experience of the EPAD study and were motivated to remain in the panel. Scotland is a unique example in this group as it was initially established as an Edinburgh based panel and had since expanded on the advice of the panel members to include participants from all Scottish centres.

The initial meetings of each panel involved similar agendas set by EPAD staff, with setting the scene and explaining the purpose of the panel, establishing rules of engagement around confidentiality and terms of reference for the panel, and nominating a participant as chair of the panel. At the Barcelona and Toulouse panels, a vice-chair was also selected to support with the leadership of the panel.

### Logistics of running the panel

The panels were all set up to run twice a year, with ad-hoc contact in between for matters arising that are time sensitive. The Barcelona panel met up to 4 times a year on the request of the participant panel members. Numbers of panel members ranged from 7 at Amsterdam to 12 in the Scottish panel, with the group size aimed to be large enough to capture a diversity of experience and opinions, whilst remaining small enough to allow everyone time to meaningfully contribute to the meetings. EPAD study staff were in attendance at every panel meeting, and in most centres the Chief Investigator or Principal Investigator also attends. The staff attended to organise the logistics of the meeting, provide study updates and answer specific questions from the panel, facilitate discussions if required and to minute the meetings. Panels met at locations convenient for participants, travel expenses were provided alongside refreshments, be that coffee or lunch depending on the preferred time of the meeting at each centre. Communication varied between countries depending on formats allowed under data protection laws, and included email, post and closed WhatsApp groups.

### Content of panel discussions

Panel meeting agendas were developed by the participants, led by the panel chair. Using the Scottish panel as an example there were standing agenda items including dementia moments (recent news stories about brain health and dementia), an update on the study progress to data (both internationally and for Scotland) and the proof of concept trials. Additional topics were then added to each meeting as desired by the participants. Additional topics discussed by panels included sustainability and longevity of the project, feedback on the study visit (including experiences, practicalities and logistical aspects), documentation review and discussions around receiving results on potential risk factors discovered through the EPAD study.

### Study advocacy and engagement

Panel members have attended a variety of events in every country to speak about their involvement with EPAD and contribute to meetings based on their experiences both as participants and as panel members. These include the IMI Stakeholder Forum 2017 where a participant represented the EPAD study on a panel discussion on PPPI, National Research Scotland (NRS) annual meeting in Perth 2018 where two panellists co-authored a poster about the panel, the EUPATI (European Patients Academy) 2018 meeting where two panellists spoke about their involvement in EPAD, and co-hosting a webinar to discuss the set up and running of a participant panel to support other centres considering hosting a panel. The Scottish and English panels have both contributed to the annual EPAD conferences held in these countries where all centre staff gather to share experiences with the study. The Barcelona panel are credited by centre staff with raising the profile of Alzheimer’s disease research in the Catalonia region through their outreach activities.

### Review of study documentation

Review of study documentation, including the study website, has been an important role played by the panels, helping to ensure any information provided to participants is understandable and appropriate for use. Suggestions from the panel led to rewording of study documents, improving readability and adapting images used in videos. The feedback received on study documentation was described as ‘positive and constructive criticism’ by staff. Many of the panels discussed protocol amendments for their advice on local implementation of changes and how best to communicate this to participants. One of these changes included the use of animated videos to support the consent process, which was a new method of communication for most sites. By discussing amendments such as this with the panel, centre staff felt confident in the protocol amendment roll-out across the centre.

The Barcelona centre has developed videos with the panel focusing on the mandatory lumbar puncture procedure in the study protocol. Perhaps unsurprisingly, participant panel members in most countries discussed experience of lumbar punctures and suggested improvements for the information provided about these. From these discussions the Spanish panel worked with the Barcelona centre to develop two videos about the procedure, one describing the procedure and one providing volunteers personal experience of undergoing the procedure. Participants were able to watch these videos prior to the procedure as a communication mechanism to support with the learning about the procedure, with staff noting a a reduction in pre-procedural nerves for some participants. This was a technique similarly used in the UK based Deep and Frequent Phenotyping pilot study where an explanatory video was produced in collaboration with the Alzheimer’s Society [32, 33]. In Amsterdam, the panel initially fed back that they often did not know whom they were seeing during their visit, as the complex procedures required a large number of staff to successfully deliver the study. Following this the team introduced a ‘study card’ to better explain the logistics of the visit and the roles of the EPAD team members involved, with the panel members collaborating on the wording and presentation of this card. This simple communication tool aimed to ensure that participants have the knowledge they want and need about their study visit, improving their overall experience at the centre.

### The study experience

Panel members discussed their recent study experiences during their local panel meetings, which led to a number of changes being enacted at centres. Some of the changes were relatively minor, such as an improvement in signage to one of the Scottish centres. Communication between researchers and participants was a common theme with advice sought on how people wanted to receive abnormal results discovered during the study (such as abnormal blood pressure, blood results or magnetic resonance imaging (MRI) findings of clinical significance). The preference from panel members was for this to be done with a personal connection, either over the phone or face to face, as opposed to receiving a letter or email with the results. As such this method of feedback for clinically significant results was implemented. Another communication change related to collection of saliva samples, which involved a relatively complex home collection method. After expressing some difficulties with this collection sites were able to spend more time discussing the instructions with the participants and suggesting helpful reminders such as setting alarms on a mobile phone for when the sample should be collected.

While the study as a whole did not routinely communicate the results of cerebrospinal fluid (CSF) tests to healthy participants, in Amsterdam the participant panel advocated for disclosure of the CSF data on the explicit request of the participant. This led to the development of standard operating procedures to support with this, and to the disclosure of amyloid status to 20 participants based at the VuMC centre.

### Study planning

Participant panel members from Scotland and Amsterdam were invited to participate in a meeting to plan the future of the EPAD study following the end of the initial funding period. Prior to this meeting members of the EPAD consortium (EPAD sites, academic and industry partners) had completed a survey to outline their views on what EPAD should prioritise to continue in the next stage of the project. During the meeting these survey responses were reviewed and a guidance document produced to advise the EPAD Management team on the next steps they should take and the possible outcomes of how the study could function in the next phase. Panel members were asked to attend to give a voice to the participant experience and add their views to how EPAD should evolve. Their views contributed to highlighting areas of convergence and divergence between participant and researcher perspectives, and to reinforcing the importance of maximising long-term sustainability of the study biobank and database.

### Participant panel perspective

Members of the Scottish and Spanish participant panels were asked by a panellist co-author to reflect on their experiences of the panel and provide feedback for use in this evaluation of the panel successes. All other panels were able to get input from at least one participant panel member. These experiences are reported below.

Prior to joining the panel, members had few expectations of what joining might mean, and there was some doubt about how much *‘influence the participants would have on the day-to-day workings of EPAD’*. People did anticipate that the meetings would be forums to ‘*provide feedback on our EPAD experiences’* and ‘*the chance to get to know other participants and to share experiences with them’*. Participants thought the panel would offer an avenue for collaboration and to spread the word about both EPAD and Alzheimer’s disease. Panel members were often motivated to join the panel by their personal experiences of living with parents with dementia.

Considering the set-up of the meetings, panel members felt there was a *‘nice balance’* of a structured approach that remained ‘*flexible as the agenda is set by the participants in conjunction with EPAD staff’.* Members appreciated the attendance of staff members who *are ‘aware of the items on the agenda … and know the outcome of each discussion’*. They report the meetings as *‘inclusive’*, with a pleasant working atmosphere, and the Scottish group in particular note that ‘*the fact that [the Principal Investigator] takes the time to come to meetings is hugely empowering’*. These experiences demonstrate the importance of providing resources to participant groups to ensure efficient operation and maintaining an informal and flexible meeting style to encourage all participants to voice their experiences and opinions. In some centres participants advocated for more regular meetings. This led to conflicts between the ambitions of the panel and desire for increased regularity of meetings, against the limits of resources the research team have to allocate. The Barcelona team had been able to support an increase in regularity of meetings, whilst other centres maintained a six-monthly schedule. Other centres were able to discuss the limitations of resource and agree with the panel that meeting twice a year would be acceptable.

Panel members reflected staff views that their input had helped to improve the participant experience by providing *‘a forum for participants to have their concerns voiced and attended to*’ which has made the yearly visits to study centres *‘as comfortable as possible*’. Importantly the panel members were key decisions makers in *‘the decision to have a Scottish panel rather than a participant panel for each Scottish trial delivery centre’.* By combining the collective experiences from these centres it is likely the panel has been able to have a bigger impact than that of four individual panels. They felt that it is clear that the Scottish Participant Panel *has ‘done a great deal to publicise the study, to disseminate the Scottish experience and to learn from what’s happening in other countries’*. The panel has managed to solidify participants as stakeholders in EPAD, by *‘reinforcing (sic) their importance in the scheme of things’* and demonstrating the *‘humanity of EPAD’*. Participant panel members felt they had contributed to *‘the transition from EPAD to EPAD2’,* which relates to sustainability discussions about the long term goals of the project, in which the participant voice has been invaluable. Panel members reported attending numerous events but by meeting other panel member groups from across Europe at the EPAD General Assembly, the groups *‘had found a voice’.* One panel has the slogan *‘we want to be part of the solution’* and this ethos is clearly reflected in many of the EPAD participant panels.

Discussions were held in panels to develop strategies to make sure *‘everyone’s voices [are] heard by the research team’*, not just those who were panel members. As part of this the Scottish group set up a satellite participant led group in Aberdeen which fed into the country wide panel.

## Discussion

The EPAD participant panel model proved beneficial to the set-up, running and future of the EPAD project, including participants as key stakeholders in the research. A key feature of the participant panels has been that they aim to be both participant-centred and, wherever possible, participant-led. Thus, the aim has been to create spaces for participant involvement and to establish the remit and scope of this involvement through ongoing dialogue between researchers and participants. The panels were established using overarching terms of reference that mandated meetings that were participant-led and held at least twice a year. Each centre then developed and adapted the set-up in line with the needs and expectations of local panel members. Overall, the panels have many similarities, with meetings chaired by a participant member and EPAD staff in attendance to organise and minute meetings. A member of staff at each site was responsible for liaising with the panels, with the EPAD ethics group co-ordinating the central panel meeting. Some differences arose in how regularly panels meet and how panels communicate between face to face meetings. The panels have been able to achieve success in affecting how the study is run in the local centres, provide support on documentation, advocate for the study at local, national and international meetings, and provide opinion on the future directions that EPAD should take.

### Strengths

We believe this nested participant panel model is adaptable for use in multi-national cohort settings. PPI can often focus on involving people living with a particular disease, who often are not directly enrolled as participants in the research project [34]. This works well in these areas because there is an identifiable group of people to approach as PPI members. However when we consider long-term research with the general population, or even ‘at risk’ groups, we need to be more creative in recognising both the commitment that individuals make to such research, and the experience and knowledge they acquire through participation. Our experience suggests though, that such panels benefits from clear responsibilities for planning and outlining the reasons for participant involvement activities, the availability of sufficient budget and a common but adaptable framework that allows expectations to be established and managed [34].

Benefits of participant involvement in the EPAD study have been reported by both panel members and researchers. Participant panel members felt they had a voice as part of the research team by being involved in panel activities, and that this was an empowering experience. Empowerment has been identified as an important, but poorly reported, potential outcome of PPPI work [35] and it is encouraging to see panel members reporting this. Panel members reflected that they had been able to influence EPAD to make study visits as comfortable for fellow participants as possible, felt they had contributed to the study by discussing it in public facing forums, and had introduced a reminder of the lived experience of research participation when study leadswere considering strategic decisions about the next steps for the EPAD project. Minute taking in meetings, and the identification of clear tasks and actions for members of the research team, enabled the panels to have meaningful and traceable impact on the development of the study, created accountability within the local study centres and helped prevent involvement becoming ‘tokenistic’. This was further facilitated by the identification of individuals within the research team at each centre responsible for liaising with the participant panel.

By involving participants, rather than interested members of the public, EPAD benefited from ongoing advice given by participants with direct experience. This has meant other participants in the study were also able to benefit in the immediate term, as opposed to just future participants potentially benefiting from participant panel advice. The panels influenced the design and impact of the research, which INVOLVE suggest contributes to the quality of the study [17]. Panel feedback on the experience of taking part in the EPAD study, and interest in the extent to which their experience was representative of the wider study population contributed to the development of a mixed-methods sub-study of participant experience across the EPAD centres. This study is currently in analysis and write-up.

### Challenges

There are, however, challenges to be considered by researchers when establishing panels. Due to the time spent setting up the panel and learning from the establishment of the PREVENT Dementia panel, the EPAD panels have largely managed to avoid these pitfalls.

Challenges at the central panel level have included the time commitment of participants and resourcing of travel for non-research staff to attend annual meetings held across Europe and the challenge of explicitly incorporating a participant role into pre-defined governance structures. Ensuring sufficient budget is available for PPI activities has been recognised as a key consideration for success [34, 36] [INSERT REF], and this is also the case for participant involvement activities.

The panels have not always moved in line with initial researcher expectations. For example, while the initial plan for participant representation included representation on study governance committees, and provision was made for this, participants at central panel meetings concluded that involvement in the General Assembly as part of the EPAD research community, and direct interaction with study leads through the local panels, provided a sufficient level of input into overall and local project decision-making.

The participant-led nature of the panels created the potential for conflicts between the ambitions of the panel and the realities of what the research staff can deliver. Others have noted the importance of identifying and addressing differences in assumptions between what staff want to achieve and what PPPI members want to achieve [37]. EPAD panels encouraged open and transparent dialogue during panel set-up and throughout the involvement process, with discussions on what actions could and would or would not be taken, with discussion of the reasons. Establishing these expectations helped with panel cohesion and understanding of role panel responsibilities among study researchers.

Structured training was not provided to either panel members or the staff facilitating panels. On reflection this is an area which we would develop in future work, to ensure all parties had a shared skill set and understanding of the participant involvement process. Training sessions for staff would include developing a shared view of the value of a shift to an equal relationship, the importance of reducing the use of technical language and how to recruit participant panel members, as identified in previous studies of PPI in UK clinical trials [38]. Training for panel members could focus on adapting their skill set to the research context as suggested in INVOLVE training support guidelines [39]. Again, however, it is important to consider the challenges associated with delivering such training across countries, in multiple languages, and the resources associated with this.

Particular challenges related to the area of research and the scope of the study. The first was the recruitment of a group of participants across the diagnostic spectrum involved in the cohort study, which recruited both healthy volunteers and people with mild cognitive impairment. In the panel the aim was to capture a variety of voices to represent the spectrum of experiences in the EPAD study. However, the panel members at all centres were for the most healthy volunteers rather than participants with mild cognitive impairments. One of the contributors to this imbalance was that the original participant recruitment to the study was biased towards healthy volunteers and as such when the panels were established the majority of participants invited to join were cognitively healthy. As such there was a group of participants in the EPAD LCS cohort who were currently not well represented in the panel memberships. Reasons for this difficulty in engagement, reported by the staff involved in supporting the panels, include both researcher bias about burdening patient participants, participant confidence in attending an unfamiliar environment and logistics of attending for someone who may prefer to have a study partner with them. Other than cognitive impairment the panels reflected the study participants well, with diversity reflected in multiple European panels. We also note that the aim of PPI work is not to have a representative sample, rather to have input from participants who reflect the diversity of the cohort. Future work in this area should consider ways to tackle inequity of diversity of cognitive impairment in participant representation so as to ensure equality with regard to actual involvement [40].

Data gathered for this paper was done in an informal manner after staff reflected that the panels had been successful and sharing the story may benefit others. While we feel that we have been able to provide a good overview of the panels, we did not have pre-set objective measures of success against which to measure the panels. Setting up clearly defined, achievable, measurable outcomes from the start may help to monitor the success of planned participant involvement activities. This would support development of an evidence base that is data driven, which is currently lacking as shown by recent systematic reviews [41]. However, in a context that aims to be participant-led and multinational, it is also important that any such evaluation is both responsive and adaptable to both participant values and the place of involvement activities in local research practice and governance frameworks.

### Recommendations

We believe the model employed in the EPAD study is adaptable to other projects, both at international levels and with other long-term research populations. We have developed a series of recommendations for other researchers below.

Recommendation One: Develop central terms of reference to establish core values and goals across all panels, but allow local adaptations to foster participant ownership of their group.

Recommendation Two: Empower and support participants to chair meetings and use staff in support roles, to ensure the meetings are participant-led but not burdensome for participant members from an organisation perspective.

Recommendation Three: Ensure sufficient budget is allowed for participant involvement activities.

Recommendation Four: Identify training needs, both for participant members and staff, to support engagement with the participant panel.

Recommendation Five: Establish how outcomes for participant involvement will enable success to be monitored against the goals established by participants.

## Conclusion

The EPAD participant panel model has shown that large scale participant involvement can be successfully conducted in a multi-centre, pan-European study, working across multiple languages, and with the goals and direction of involvement led, in large part, by participants themselves. While such a participant-led approach can present challenges to both researchers and participants, it ensures that the panels are reflective of and responsive to, the needs and concerns of participants themselves. We believe this is a model that can be adapted to suit similar study populations.

## Supplementary information

**Additional file 1.** GRIPP2 Short form

## Data Availability

Data sharing is not applicable to this article as no datasets were generated or analysed during the current study.

## Abbreviations

EPAD: European Prevention of Alzheimer’s Dementia
AD: Alzheimer’s disease
LCS: Longitudinal cohort study
PoC: Proof of concept
PPI: Patient and public involvement
PPPI: Patient, public and participant involvement
