# Supplementary material for: Involving research participants in a pan-European research initiative: the EPAD participant panel experience
Source: Res Involv Engagem. 2020 Oct 15;6:62. doi: 10.1186/s40900-020-00236-z (PMC7566117; doi:10.1186/s40900-020-00236-z)
Supplement: Supplementary file 1 — Additional file 1. GRIPP2 Short form [file 40900_2020_236_MOESM1_ESM.pdf]

INVOLVING RESEARCH PARTICIPANTS IN A PAN-EUROPEAN RESEARCH INITIATIVE: THE EPAD PARTICIPANT PANEL EXPERIENCE: GRIPP2 Short form

**Section and topic**	**Item**	**Reported on page No**
1: Aim	Report the aim of PPI in the study	3
2: Methods	Provide a clear description of the methods used for PPI in the study	3-6
3: Study results	Outcomes—Report the results of PPI in the study, including both positive and negative outcomes	6-9
4: Discussion and conclusions	Outcomes—Comment on the extent to which PPI influenced the study overall. Describe positive and negative effects	9-11
5: Reflections/critical perspective	Comment critically on the study, reflecting on the things that went well and those that did not, so others can learn from this experience	11
